# Hyponatremia is a marker of disease severity in HIV-infected patients: a retrospective cohort study

**DOI:** 10.1186/s12879-017-2191-5

**Published:** 2017-01-26

**Authors:** Philippe Braconnier, Marc Delforge, Maria Garjau, Karl Martin Wissing, Stéphane De Wit

**Affiliations:** 1 Department of Infectious Diseases, AIDS Reference Center, Saint-Pierre University Hospital, Université Libre de Bruxelles, Brussels, Belgium; 2 Department of Nephrology, Universitair Ziekenhuis Brussel, Vrije Universiteit Brussel, Brussels, Belgium; 3 Department of Nephrology, Centre Hospitalier Universitaire Brugmann, Brussels, Belgium

**Keywords:** Acquired immunodeficiency syndrome, Human immunodeficiency virus, Hyponatremia, Mortality, Risk factors

## Abstract

**Background:**

Hyponatremia is a frequent electrolyte disorder in HIV-infected patients with a prevalence of up to 56% in the pre-cART era. Several studies have demonstrated that patients with hyponatremia are at an increased risk of death. We aimed to investigate the prevalence of hyponatremia in the recent cART-era and evaluate its association with mortality.

**Methods:**

Single-center retrospective cohort study. A total of 1196 newly diagnosed and cART-naïve HIV patients followed at the AIDS Reference Center, St Pierre University Hospital in Brussels, Belgium, between 1 January 1998 and 31 December 2013 were included. Hyponatremia was defined as a baseline natremia lower than 135 mmol/l. The outcome of interest was the occurrence of death.

**Results:**

In this study 177 (14.8%) patients had hyponatremia at baseline with a median natremia of 132.0 mmol/l [interquartile range (IQR) 130.0-134.0 mmol/l]. Hyponatremic patients had a lower CD4 cell count (207.5 ± 197.7/μl vs 400.4 ± 277.0/μl; *P* < 0.0001) and a higher prevalence of AIDS (50.3% vs 12.4%; *P* < 0.0001) compared to normonatremic patients. A significantly higher proportion of patients with hyponatremia were hospitalized at first contact (72.3% vs 20.0%; *P* < 0.0001). During the follow-up hyponatremic patients had a shorter median time to a first hospitalization (2.0 IQR [0.0-12.0] months vs 13.0 IQR [2.0-29.0] months; *P* = 0.001) and an increased incident hospitalization rate (785/1000 patient-years, 95% CI 725–845 vs 370/1000 patient-years, 95% CI 352–388; *P* < 0.0001]. The incident mortality rate was 28.3/1000 patient-years (95% CI 18.15-42.16) in patients with hyponatremia compared to 9.33/1000 patient-years (95% CI 6.63-12.75) in normonatremic patients (*P* < 0.0001). Three-year cumulative survival rates were 85.8% ± 3.0% in hyponatremic patients and 96.3% ± 0.7% in normonatremic patients (log-rank *P* < 0.0001). However, in a multivariate Cox model adjusting for other risk factors such as AIDS, CD4 count < 350/μl and hepatitis C, hyponatremia was no longer a predictor for patient death (hazard ratio: 1.03, 95% CI 0.54-1.97; *P* = 0.935).

**Conclusions:**

Hyponatremia is a marker of severity of HIV-disease but not an independent risk factor for mortality. HIV-patients with a low serum sodium at baseline might benefit from a close follow-up to improve outcomes.

**Electronic supplementary material:**

The online version of this article (doi:10.1186/s12879-017-2191-5) contains supplementary material, which is available to authorized users.

## Background

Hyponatremia, commonly defined as a serum sodium concentation < 135 mmol/l, is the most common electrolyte abnormality encountered in clinical practice. In the general population its prevalence is estimated 4–7% in an ambulatory setting and 15–30% in hospital care [1, 2]. Several studies have shown that hyponatremia is associated with an increased risk of death both in hospitalized and in ambulatory patients [3, 4]. This has classically been demonstrated in patients with heart failure [5] and cirrhosis [6] where hyponatremia seems to reflect the severity of the underlying disease, but recent data suggest that chronic and mild hyponatremia in the general population is also associated with mortality [7]. Several conditions may predispose to the development of hyponatremia in human immunodeficiency virus (HIV)-positive patients: severe volume depletion which is most often caused by gastrointestinal fluid losses such as diarrhea; the syndrome of inappropriate antidiuresis usually due to drugs (most common antidepressants, anticonvulsants but also pyrazinamide, ethambutol) [8], malignancy, pulmonary and central nervous system infections (such as Pneumocystis jiroveci infection, neurosyphilis or toxoplasmosis) [9, 10]; and adrenal insufficiency which is a less common cause.

In HIV-infected patients with the acquired immunodeficiency syndrome (AIDS) or AIDS-related complex (ARC) a prevalence of 35–56% of hyponatremia has been observed in several studies performed before the era of combined antiretroviral therapy (cART) [9, 11, 12]. Tang et al. demonstrated in a prospective study of 212 in-patients with AIDS or ARC that on average hospitalizations lasted 8 days longer in those with hyponatremia than in those with normal serum sodium concentrations and that patients in the hyponatremic group had nearly two-fold higher risk of death [12]. In the pre-cART era hyponatremia in ambulatory HIV-positive patients had also been reported to be associated with a reduction of median survival from 39 months to 11.5 months as compared to HIV-positive patients without hyponatremia [13].

In 1997 the introduction of the combined antiretroviral therapy resulted in a 60–80% decline of AIDS, mortality and hospitalisations in regions were these medications have been made available to patients [14]. Since then little is known about the prevalence of hyponatremia and its association with mortality in HIV patients. Although incidences of hyponatremia in HIV patients were previously high and strongly associated with an increased risk of death, treatment has changed dramatically with possible effects on the association of hyponatremia with mortality. The aims of our study were to investigate the prevalence of hyponatremia in the more recent cART-era in a single-center cohort of HIV-positive patients with long term follow up and to provide a detailed description of health conditions associated with hyponatremia which might act as confounders of the association between hyponatremia and death. The hypothesis of our study was that, similar to the general population, hyponatremia is an independent risk factor for death of HIV-positive patients after adjusting for confounding risk factors.

## Methods

### Study design and participants

We conducted a retrospective cohort study including all newly diagnosed and cART-naïve HIV patients followed at the AIDS Reference Center St Pierre University Hospital in Brussels, Belgium, between 1 January 1998 and 31 December 2013. During this period a total of 4195 patients entered the Brussels St Pierre Cohort. We excluded 2999 patients: 1247 non cART-naïve patients and 1752 patients without available baseline natremia. A total of 1196 patients were included in the study and initiated on cART during the follow up period. The Brussels St Pierre Cohort collects information about all the adult HIV-infected patients (≥18 years) ever seen at St Pierre University Hospital in Brussels for consultation or for hospitalization. Types of data collected include demographic, therapeutic and laboratory data, clinical events and health services utilization. The follow-up interval for data collection including CD4 and viral load determination is every 3 to 4 months. Prospective data are collected through structured questionnaires and medical records. Data quality is assured by an automatic check of data validity through the data system and data validation by a physician. The Brussels St Pierre cohort participates in the DAD, EuroSIDA and COHERE cohorts and has been validated by numerous publications [15–17].

### Data definition

Exposure of interest: Hyponatremia was defined as a serum sodium concentration lower than 135 mmol/l and was used to divide the population in two exposure categories: hyponatremic patients and normonatremic patients (natremia ≥ 135 mmol/l). The serum sodium concentration was measured by indirect ion selective electrode potentiometry (Roche) and recorded automatically at baseline, the first available laboratory value at patient inclusion in the cohort. Common treatment strategies for hyponatremia in our clinic are etiology-based and include restricting fluid intake, hypertonic salt administration and increasing solute intake with urea or a combination of low-dose loop diuretics and oral sodium chloride.

### Collection of potential confounding variables

The following demographic data were extracted for all included patients: age, gender, ethnicity (black, caucasian, other) and sexual orientation (heterosexual or homo-/bi-sexual). All demographic, clinical and laboratory data were baseline values recorded by a data manager at patient inclusion in the cohort and validated by a physician. CD4 cell count nadir was defined as the lowest CD4 cell count (/μl) measured prior to patient inclusion in the cohort. The AIDS stage at inclusion was defined according to the criteria of the Centers for Disease Control and Prevention [18]. A serum hemoglobine ≤ 14 g/dl for men and ≤ 12 g/dl for women defined anemia. Results from the EuroSIDA study group have previously reported that anemia is a strong independent prognostic marker for death in HIV-infected patients from across Europe [19].

Hyperlipidemia was defined according to the National Cholesterol Education Program (NCEP) Guidelines (total cholesterol ≥ 200 mg/dl or LDL ≥ 130 mg/dl or triglycerids ≥ 150 mg/dl) [20] and by the intake of lipid-lowering drugs (statines or fibrates). Patients were considered diabetic in case of insuline therapy, oral antidiabetics or HbA1c ≥ 6.5% [21]. The presence of a hepatitis B surface antigen defined active hepatitis B disease. Patients were considered hepatitis C positive if anti-hepatitis C antibodies were present. An analysis of data from the CASCADE collaboration demonstrated that individuals co-infected with HIV and hepatitis C have a higher risk of death from HIV or liver disease than patients infected with only HIV [22]. As a validated indirect marker of liver fibrosis the FIB-4 index was calculated according to the following formula: FIB-4 = age (years) × AST [U/L]/(platelets [10^9^/L] × (ALT [U/L])^1/2^) [23]. A cut-off > 3.25 was used because it predicts a significant liver fibrosis with a positive predictive value of 65% and a specificity of 97% [23]. The FIB-4 index has recently been shown to be an independent predictor of liver-related death in HIV infected patients initiating antiretroviral therapy [24].

### Follow up period

During the 15-year follow up the characteristics of the patient population and prevalence of hyponatremia were likely to change. We therefore subdivided the observation period into two succcessive sub-periods (1998–2004 and 2005–2013) and tested whether these were associated with the exposure and outcome of interest.

### Outcomes of interest

The occurence of death in the follow-up period was the outcome of interest. The CoDe Coding Causes of Death (CoDe) in HIV protocol was used as a classification system for the causes of death (http://www.cphiv.dk/Tools-Standards/CoDe/) which were subdivided in HIV-related and non HIV-related deaths. A secondary outcome was the number of hospitalizations during the follow-up period. Causes of hospital admission were divided into 20 categories, which were the most commonly reported in the scientific literature [25]: AIDS-related, malnutrition/wasting, parasitic infections, bacterial infections, malignancies, drug toxicities, neurological, cardiovascular, renal, endocrine/metabolic, haematological, respiratory, digestive, liver, viral, skin/soft tissue, psychiatric disorders gynaecological, other causes or unknown. Patients were considered lost to follow-up in case of no contact for more than one year.

### Statistical analysis

Patient characteristics were described using mean and standard deviation or median and interquartile range (IQR) for continuous data, and frequencies with percentages for qualitative data. Hypothesis testing for differences between the two exposure categories was done with the Mann–Whitney *U* test for continuous data and the Chi-square test or Fishers exact test for categorical data. In case of missing data only the available data was analyzed. Patient survival was estimated with the Kaplan-Meier method and the null hypothesis of equal survival in the two exposure categories tested using the log-rank test. Mortality was quantified as the incidence death rates per 1000 patient-years at risk. Time at risk was defined as the delay between inclusion in the cohort and patient death. Patients were censored in case of loss to follow-up or at the end of the study period on December 31, 2013. As many patients were hospitalized multiple times, we also calculated the incidence rate of hospitalization without censoring the patient after the first hospitalization. A negative binomial regression was used to test the association.

The effect of hyponatremia and other patient characteristics associated with baseline hyponatremia on patient death was first investigated by univariate Cox proportional-hazards regression. A multivariable Cox model was then build by including all covariates that were significantly associated with patient death in the univariate analysis. The assumption of proportional hazards over time was verified and met by all covariates introduced in the models. We have generated time dependent covariates by creating interactions of the predictors and a function of survival time included in the model. If any of these time dependent covariates were significant then those predictors were not proportional which was not the case in our model. A type-1 error of <0.05 was used to reject the null hypothesis for all hypothesis testing. Analysis and graphs were produced using SAS statistical software (version 9.4; SAS Institute, Cary, North Carolina, USA).

### Ethical considerations

The procedures of data collection and measures taken to maintain data confidentiality in the database of Brussels St Pierre cohort have been reviewed and approved by the Hôpital St Pierre Ethics committee. Patients consented at the time of inclusion in the cohort that their medical data would be collected in the centre and European databases for medical and research purposes.

## Results

### Baseline patient characteristics

In total 1196 HIV-infected patients were included in the cohort with a mean age of 36.8 ± 10.7 years and a predominance of men (62.8%) (Table 1; Title: Main characteristics of hyponatremic and normonatremic patients).

**Table 1:** Main characteristics of hyponatremic and normonatremic patients

Characteristics	Total (*N* = 1196)	Na < 135 mmol/l (*N* = 177)	Na ≥ 135 mmol/l (*N* = 1019)	*P*-value
Age (years)^a^	36.8 ± 10.7	37.4 ± 10.0	36.7 ± 10.9	0.238
Female gender (n)	445 (37.2%)	90 (50.8%)	355 (34.8%)	<0.0001
African ethnicity (n)	612 (51.2%)	125 (70.6%)	487 (47.8%)	<0.0001
Homo-bisexual orientation (n)	395 (33.0%)	26 (14.7%)	369 (36.2%)	<0.0001
Natremia (mmol/l)^b^	139.0 (136.0-141.0)	132.0 (130.0-134.0)	139.0 (137.0-141.0)	<0.0001
Hospitalization at first contact (n)	332 (27.7%)	128 (72.3%)	204 (20.0%)	<0.0001
Acquired immunodeficiency syndrome (AIDS) (n)	215 (18.0%)	89 (50.3%)	126 (12.4%)	<0.0001
CD4 cell count (/μl)^a^	371.8 ± 275.3	207.5 ± 197.7	400.4 ± 277.0	<0.0001
CD4 cell count < 350/μl (n)	611 (51.1%)	143 (80.8%)	468 (45.9%)	<0.0001
CD4 nadir (/μl)^a^	362.8 ± 267.6	200.8 ± 184.1	391.2 ± 269.9	<0.0001
CD4 nadir < 200/μl (n)	360 (30.1%)	106 (59.9%)	254 (24.9%)	<0.0001
HIV viral load (copies/ml)^b^	71600 (13500–100000)	100000 (55000–313500)	58750 (11800–100000)	<0.0001
HIV viral load > 100 000 copies/ml (n)	465 (38.9%)	104 (58.7%)	361 (35.4%)	<0.0001
Hepatitis B (n)	69 (5.8%)	14 (7.9%)	55 (5.4%)	0.223
Hepatitis C (n)	69 (5.8%)	12 (6.8%)	57 (5.6%)	0.478
Fib 4 score > 3.25 (n)	77 (6.4%)	28 (15.8%)	49 (4.8%)	<0.0001
Anemia (n)	597 (50%)	138 (78%)	459 (45%)	<0.0001
Hyperlipidemia (n)	418 (34.9%)	58 (32.8%)	360 (35.3%)	0.550
Mean triglyceridemia (mg/dl)^a^	111.1 ± 68.5	132.4 ± 79.9	108.0 ± 66.0	<0.0001
Mean estimated glomerular filtration rate^c^ (ml/min)^a^	112.3 ± 23.3	111.4 ± 29.3	112.5 ± 22.0	0.227
Diabetes mellitus (n)	52 (4.3%)	14 (7.9%)	38 (3.7%)	0.0254
Antihypertensive drugs (n)	39 (3.3%)	4 (2.2%)	35 (3.4%)	0.645

^a^mean ± standard deviation

^b^median (interquartile range)

^c^estimated using CKD-EPI equation [36]

Hyponatremia at baseline was present in 177 (14.8%) patients with a median serum sodium concentration of 132 (IQR130-134) mmol/l. The hyponatremic patient group had significantly different demographic characteristics: more patients were women and from African descent with a lower prevalence of homo-bisexual orientation.

A significantly higher proportion of patients with hyponatremia were hospitalized at first contact [72.3% versus (vs) 20.0%]. In comparison with people without hyponatremia, those with hyponatremia had significantly more severe HIV disease with a lower CD4 cell count (208 ± 198/μl vs 400.4 ± 277/μl), about twice the HIV viral load and an approximatively four-fold higher prevalence of AIDS. No significant differences in the co-existence of a hepatitis B or C infection were noted between the two groups but hyponatremic patients had more frequently a high FIB-4 index (>3.25) suggestive of a more prevalent liver fibrosis.

**Fig. 1:**
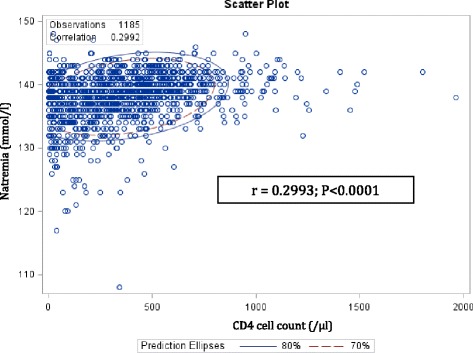
Scatter plot of natremia and CD4 count. A decrease in serum sodium is positively correlated with a decreasing CD4 cell count (Pearson Correlation Coefficient = 0.2993; *P* < 0.0001)

**Fig. 2:**
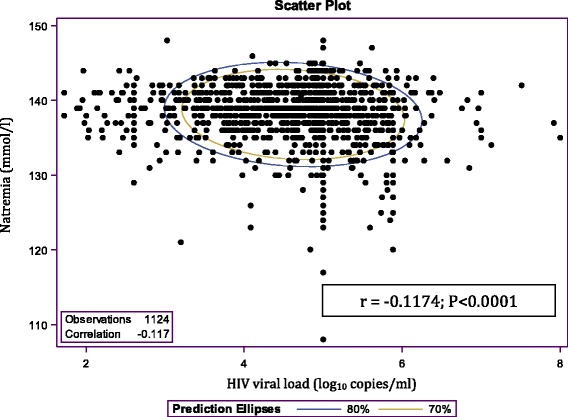
Scatter plot of natremia and HIV viral load. A decrease in serum sodium is negatively correlated with an increasing HIV viral load (Pearson Correlation Coefficient = −0.1174; *P* < 0.0001)

Baseline serum sodium concentration as a continuous variable was also associated with CD4 cell count (Fig. 1). A decrease in natremia was positively correlated with a decreasing CD4 cell count (Pearson Correlation Coefficient = 0.2993; *P* < 0.0001). Furthermore, natremia correlated negatively with the HIV viral load (Pearson Correlation Coefficient = −0.1174; *P* < 0.0001; Fig. 2).

### Association of hyponatremia with hospitalization rate and mortality

About 70% of patients were started on cART after inclusion in the cohort. The median follow-up time was 36.0 (IQR 9.0-80.0) months and the incidence of patients with loss to follow-up was 8.2 [95% confidence interval (CI) 7.5-9.1] per 100 patient-years (Table 2; Title: Outcomes in hyponatremic and normonatremic patients).

**Table 2:** Outcomes in hyponatremic and normonatremic patients

Characteristics	Total (*N* = 1196)	Na < 135 mmol/l (*N* = 177)	Na ≥ 135 mmol/l (*N* = 1019)	*P*-value
Combined antiretroviral therapy (n)	845 (70.6%)	130 (73.4%)	715 (70.2%)	0.421
Follow-up (months)^a^	36.0 (9.0-80.0)	41.0 (4.0-115.0)	35.0 (10.0-77.0)	0.554
Loss to follow-up/100 patient-years (95% CI)	8.2 (7.5-9.1)	8.4 (7.4-9.1)	8.2 (6.5-10.6)	0.870
Deaths (n)	63 (5.3%)	24 (13.5%)	39 (3.8%)	<0.0001
Death rate/1000 patient-years (95% CI)	12.5 (9.6-16.0)	28.3 (18.1-42.2)	9.33 (6.6-12.7)	<0.0001
Hospitalization rate/1000 patient-years (95% CI)	440 (422–458)	785 (725–845)	370 (252–388)	<0.0001
Mean number of hospitalizations per patient (n)^b^	2.2 ± 4.9	4.3 ± 9.5	1.8 ± 3.3	<0.0001
Median time to death (months)^a^	12.0 (3.0-34.0)	6.0 (2.0-22.5)	14.0 (4.0-39.0)	0.169
Median time to first hospitalization (months)^a,c^	12.0 (2.0-28.0)	2.0 (0.0-12.0)	13.0 (2.0-29.0)	0.0012

^a^median (interquartile range)

^b^mean ± standard deviation

^c^excluding patients hospitalized at first contact

Patients lost to follow-up were significantly younger, more frequently of female gender, hetero-sexual and of African ethnicity. They were more often hospitalized at first contact and had a shorter median time to a first hospitalisation. However, there was no significant difference in the prevalence of hyponatremia, CD4 count or AIDS (see Additional file 1).

Hyponatremic patients had a higher incident hospitalization rate of 785/1000 patient-years (95% CI 725 to 845) compared to 370/1000 patient-years (95% CI 352 to 388) in normonatremic patients (incidence rate ratio: 2.12, 95% CI 1.94-2.32; *P* < 0.0001) and a shorter median time to a first hospitalization (2.0 [0.0-12.0] vs 13.0 [2.0-29.0] months).

**Fig. 3:**
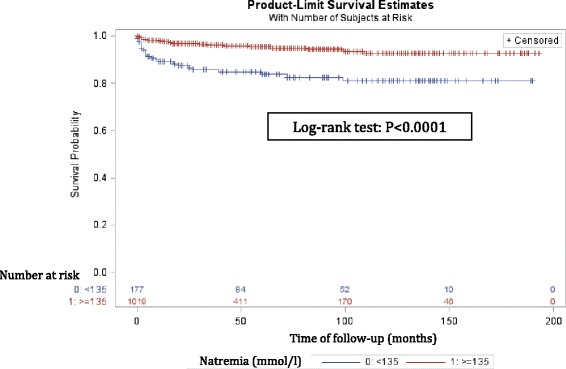
Kaplan-Meier estimates of survival by natremia group. Population at risk during the follow-up is tabulated below the graph. The 6-months, 1-year, 3-year and 5-year cumulative survival rates were 98.1% ± 0.4%, 97.8% ± 0.5%, 96.3% ± 0.7%, 95.5% ± 4.5% respectively in normonatremic patients versus 91.4% ± 2.3%, 89.2% ± 2.6%, 85.8% ± 3.0%, 83.8% ± 16.2% respectively in the hyponatremic group. Hypothesis testing by the log-rank test with *P* < 0.0001

In the overall study population, 63 patients (5.3%) died during follow up with an increased mortality for patients with hyponatremia at baseline (13.5% vs 3.8%; *P* < 0.0001). At the univariate level patients with hyponatremia had a significantly increased incident mortality rate of 28.3/1000 patient-years (95% CI 18.15-42.16) compared to 9.33/1000 patient-years (95% CI 6.63-12.75) for normonatremic patients (incidence rate ratio: 3.04, 95% CI 1.80-5.03; *P* < 0.0001). The Kaplan-Meier estimates of survival differed significantly between the two natremia groups (log-rank test: *P* < 0.001; Fig. 3). Adjusting hyponatremia for other risk factors of patient death in a multivariate Cox model reduced the hazard ratio from 3.94 to 1.03 (95% CI 0.54-1.97), close to unity and no longer statistically significant (Table 3; Title: Risk factors for mortality of patients in univariate/multivariate Cox’s proportional hazard models).

**Table 3:** Risk factors for mortality of patients in univariate/multivariate Cox’s proportional hazard models

	Univariate Model		Multivariate Model	
Risk factors	Hazard ratio (95% Confidence interval)	*P*-value	Hazard ratio (95% Confidence interval)	*P*-value
Age < 35 years	0.51 (0.29-0.87)	0.014	0.91 (0.48-1.73)	0.782
Female gender	1.68 (1.01-2.80)	0.045	1.65 (0.86-3.16)	0.132
African ethnicity	1.29 (0.77-2.15)	0.366		
Homo-bisexuel orientation	0.28 (0.13-0.59)	0.0003	0.90 (0.34-2.34)	0.827
Natremia < 135 mmol/l	3.94 (2.30-6.74)	<0.0001	1.03 (0.54-1.97)	0.935
AIDS	8.83 (5.18-15.06)	<0.0001	5.24 (2.59-10.62)	<0.0001
CD4 cell count < 350/μl	11.71 (4.66-29.43)	<0.0001	6.58 (1.89-23.06)	0.003
HIV viral load > 100 000 copies/ml	3.36 (1.87-6.03)	<0.0001	1.15 (0.56-2.37)	0.702
Hepatitis B	0.23 (0.032-1.71)	0.172		
Hepatitis C	2.54 (1.15-5.60)	0.0259	2.70 (1.172-6.23)	0.02
Fib4 score > 3.25	3.84 (1.95-7.55)	<0.0001	1.69 (0.77-3.74)	0.192
Anemia	4.30 (2.95-6.27)	<0.0001	1.15 (0.75-2.84)	0.263
Hyperlipidemia	0.93 (0.54-1.59)	0.892		
Diabetes mellitus	4.22 (1.96-9.11)	0.0011	2.07 (0.88-4.85)	0.096
Inclusion period 1998-2004	2.54 (1.52-4.25)	0.0004	1.32 (0.72 – 2.42)	0.372

AIDS at inclusion, a CD4 count < 350/μl and hepatitis C were independent predictors of patient death in the multivariate model. In a supplemental analysis the population was restricted to the 356 patients who had advanced HIV disease with a CD4 count < 200/μl. A total of 106 (29.8%) of patients had hyponatremia which was significantly associated with mortality in univariate analysis but remained non significant in the multivariate model (see Additional file 2).

### Evolution of the study population characteristics and prevalence of hyponatremia over time

Long term follow-up allowed to evaluate the evolution of hyponatremia over the last 15 years. The prevalence of hyponatremia clearly decreased over time: in the 1998–2004 period, 27.0% of the patients had hyponatremia at baseline compared to only 8.0% in the more recent period (2005–2013). Interestingly, there was a parallel evolution of mortality decreasing from 8.4 to 3.5% as well as AIDS (prevalence decreased from 23.5 to 14.9% in the recent period; *P* = 0.0003). Moreover, in the 1998–2004 period, patients were more frequently hospitalized at first contact (39.7% vs 21.2%; *P* < 0.0001) and more often admitted during follow-up (mean number of hospitalizations per patient: 3.3 ± 6.9 vs 1.6 ± 3.0; *P* < 0.0001). As the period is clearly associated with both hyponatremia and survival it might act as a confounder of the effect of hyponatremia on survival. However, the inclusion of the period as variable in the Cox proportional hazard model did not influence our results significantly (Table 3).

Demographically our patient population did also change over time: patients in the period 2005–2013 were less frequently of female gender (31.8% vs 46.9%) and African ethnicity (44.8% vs 62.7%) but significantly more of homo-bisexual orientation (40.8% vs 19.0%). Finally, the incidence of patients lost to follow up in the earlier 1998–2004 period was higher compared to the more recent period 2005–2013 (11.2 95% CI 9.0-13.8 vs 8.1 95% CI 7.0-9.3 per 100 patient-years, *P* = 0.012).

### Causes of death

About half of the patients died of an AIDS-related cause (AIDS with an ongoing active infection or malignancy). There were no significant differences between the two groups of natremia (AIDS-related deaths in hyponatremic patients: 54.1% versus 46.1% in normonatremic patients, *P* = 0.609). Four patients died from parasitic or fungal infections, 5 from bacterial infections such as tuberculosis or sepsis, 13 from AIDS-related malignancies and 3 from neurological causes in relation with AIDS (2 multifocal leucoencephalitis and 1 cerebral hemorrhage in the context of disseminated tuberculosis). Six patients died from complex associations of AIDS related infection and cancer without possibility to clearly identify the principal cause of death. Nine out of 31 deaths implicated disease of the central nervous system (29% of AIDS-related deaths). There was no difference in the number of deaths from central nervous system-related causes in patients with hyponatremia (31%) as compared to patients with normonatremia (28%; *P* = 1.000).

Other causes of death included not HIV related infection (17.5%), not HIV related malignancy (7.9%), active substance abuse (3.2%), chronic viral hepatitis (1.6%), ischemic heart disease (1.6%) or other (6.3%). The causes of death were unclassified or unknown in 4 patients (6.3%).

### Causes of hospital admission

In the overall cohort leading causes of hospital admission were bacterial infections (338 admissions, 14.6%), malignancies (206 admissions, 8.9%), respiratory (187 admissions, 8.1%), AIDS-related (134 admissions, 5.8%), viral (118 admissions, 5.1%), digestive (108 admissions, 4.7%), neurological (105 admissions, 4.5%) and parasitic infections (90 admissions, 3.9%). The cause of hospital admission was unknown in 403 admissions (17.4%). Patients with hyponatremia had an approximatively 5-fold increased risk of being admitted for an AIDS-related cause (odds ratio [OR] = 5.15, 95% CI 3.28-8.08; *P* < 0.0001) or endocrine/metabolic causes (OR = 5.07, 95% CI 1.68-15.27; *P* < 0.007); a 4.5-fold increased risk for bacterial infections (OR = 4.49, 95% CI 3.16-6.37; *P* < 0.0001); a 4-fold increased risk for respiratory causes (OR = 4.04, 95% CI 2.66-6.12; *P* < 0.0001); a 3-fold increased risk for malignancies (OR = 3.00, 95% CI 1.67-5.41; *P* = 0.0006); a 2-fold risk for drug toxicities (OR = 2.11, 95% CI 1.04-4.28; *P* = 0.045), for parasitic infections (OR = 2.05, 95% CI 1.12-3.77; *P* = 0.024), for digestive (OR = 1.86, 95% CI 1.08-3.19; *P* = 0.033) or gynaecological reasons (OR = 1.85, 95% CI 1.13-3.05; *P* = 0.018) (see Additional file 3).

### Evolution of natremia during follow up

Among patients who had hyponatremia at baseline, 19.2% of patients still had hyponatremia at last contact compared to 2.5% in baseline normonatremic patients (*P* < 0.0001). Interestingly, hyponatremia at last contact was significantly more prevalent in deceased patients than in living patients (21.3% vs 3.9%; *P* < 0.0001).

## Discussion

The present study agrees with previous reports in HIV patients that baseline hyponatremia is associated with an increased risk of mortality in univariate analysis without adjustment for confounders [26–28]. All of these studies originated from Africa or Asia and had relatively short follow up periods and higher mortality rates than observed in North American or European populations. A prospective cohort study of 661 HIV infected women in sub-Saharian Africa demonstrated that baseline hyponatremia was associated with a more than four-fold increase in the risk of death [26]. This study had a limited follow-up of one year after initiation of antiretroviral therapy in patients with a high mortality (8%) mainly due to tuberculosis (32%). More recently a prospective cohort study of 141 hospitalized patients with advanced stages of HIV infection in China showed that baseline hyponatremia was present in 28.4% of patients and independently associated with three-fold increase of death within six months after admission [27]. In this study, 72.9% of patients had advanced HIV disease [World Health Organization (WHO) stage 4] and 21.3% of patients died during the first six months of admission which is higher than the short-term mortality rates in other studies [29]. Other limitations included the small sample size (141 patients) and the short follow-up time of 6 months. Another Chinese study by Xu included 387 cART-naïve hospitalized patients with variable follow-up times from 1 week to 72 months [28]. Those patients had a high prevalence of hyponatremia (53.2%), advanced HIV disease (84.2% were WHO stage III or IV) and a low overall survival (3-year cumulative survival rates were 47.8% ± 68.5% for patients with moderate/severe hyponatremia, 59.8% ± 65.0% for those with mild hyponatremia and 78.2% ± 63.8% for normonatremic patients). Reporting of mortality in this cohort was also restricted to AIDS-related death.

Interestingly, in contrast to the previously cited studies, hyponatremia was not an independent risk factor for mortality in our analysis, even after restricting the analysis to patients with advanced HIV disease (CD4 < 200/μl). In fact, adjustement for AIDS as only covariate in the multivariate analysis completely canceled the effect of hyponatremia on mortality. This suggests that hyponatremia might be a marker of severe HIV-related disease and not directly implicated in the increased incidence rate of patient death. This is supported by the fact that patients with hyponatremia at baseline had a significantly more severe HIV disease with lower CD4 count, higher number of hospitalisations in the follow-up and increased prevalence of AIDS compared to patients with a normal serum sodium concentration (50.3 vs 12.4%). Xu and collegues have nicely demonstrated that natremia is negatively correlated with WHO stage [28]. In their study patients with advanced WHO stages had a significantly lower natremia: mean serum sodium concentration was 136.7 ± 5.4 mmol/l in patients in WHO stage I/II, 132.8 ± 6.6 mmol/l in stage III and 131.7 ± 6.3 mmol/l in stage IV (F = 14.586; *P* < 0.001). Our analysis confirms the previously reported significant positive correlation by Xu between the serum sodium concentration and CD4 cell count. Furthermore a decrease in natremia is negatively correlated with an increasing HIV viral load which is also an argument for the hypothesis of hyponatremia as a marker of severity.

It is also of interest to note that increased mortality in hyponatremic patients is mainly seen during the first 6 months of follow-up, with Kaplan-Meier survival curve thereafter showing a parallel decrease in survival between the two natremia groups. Patients with a low serum sodium at baseline have an increased mortality in the first months because of a more advanced HIV disease but on the long term hyponatremia at baseline does not appear to be predictive of mortality.

Another finding which was quite unexpected is that there were not significantly more HIV-related deaths in patients with hyponatremia than in normonatremic patients at baseline (66.7% versus 48.7%; *P* = 0.198). This supports the idea of hyponatremia as a general marker of disease severity which is not specific for HIV. In fact, the proportion of hyponatremia at last contact was much higher in deceased patients than in living patients (21.3% vs 3.9%; *P* < 0.0001). The idea that hyponatremia does not contribute directly to mortality but is merely a surrogate marker for the severity of the underlying disease is also supported by studies in patients with advanced heart failure [30] and liver cirrhosis [31]. Although hyponatremia has been associated with mortality in various settings, there is an active debate in the literature whether hyponatremia contributes directly to mortality and if so, how this effect would be mediated. Until now no study has convincingly shown this direct contribution to mortality and it also remains unclear whether correcting hyponatremia improves outcomes [32].

Our study has limitations. First, we were not able to exclude pseudohyponatremia which is a laboratory artefact that occurs when abnormally high concentrations of lipids or proteins such as gammaglobulins in the blood interfere with the accurate measurement of sodium [33]. As hypergammaglobulinemia is a frequent occurrence in HIV patients, pseudohyponatremia might be an important and likely underdiagnosed phenomenon in this patient population [34]. Pseudohyponatremia can be ruled out by the measurement of a normal serum osmolality which was not done routinely in our study population. A second limitation is that the demographic characteristics of the study population and the prevalence of hyponatremia changed over time. However, mortality rates remained stable during the entire study period without significant interaction with time, so respect of proportionality in time can be assumed. Furthermore, to adress the issue of differences in follow-up time we calculated incidence rates to take into account the time at risk. Finally, the study suffered from a large proportion of patients lost to follow-up (incidence of 8.24 per 100 person-years of follow-up) which is higher than the 3.72 per 100 person-years of loss to follow-up reported from an international observational study [35]. However, there was considerable variation in loss to follow-up among the different countries that participated in the EuroSIDA study, varying from 0.67 to 13.35. The loss of follow-up in our cohort is probably mainly explained by socio-demographic factors as patients lost to follow-up were significantly younger, more frequently hetero-sexual and of African ethnicity (61%).

This might be a source of selection bias as patients lost to follow-up may be less healthy and therefore more likely to die leading to an underestimation of the study outcome. However, patients who dropped out had no significant clinical differences regarding the prevalence of baseline hyponatremia, CD4 count and AIDS. In addition the proportion of patients lost to follow-up did not differ among those with and without baseline hyponatremia.

## Conclusion

In conclusion, this study shows that hyponatremia is a marker of the severity of HIV-disease but not an independent risk factor for mortality as suggested by previous publications. Hyponatremic HIV patients had a lower CD4 cell count, a higher prevalence of AIDS and were more frequently hospitalized at first contact compared to normonatremic patients. Furthermore, during the follow-up period patients with hyponatremia had a shorter median time to a first hospitalization and an increased incident hospitalization and mortality rate. HIV-patients with a low serum sodium at baseline might benefit from a close follow-up to improve outcomes.

## Additional files

Additional file 1: Main characteristics of patients lost to follow-up. Clinical and socio-demographic factors in patients lost to follow-up compared with patients not lost to follow-up. ^1^mean ± standard deviation; ^2^median (interquartile range); ^3^excluding patients hospitalized at first contact. (DOCX 92 kb)
Additional file 2: Risk factors for mortality of patients with CD4 < 200/μl in univariate/multivariate Cox’s proportional hazard models. Association of hyponatremia with mortality in a restricted population of patients who had advanced HIV disease with a CD4 count < 200/μl. (DOCX 52 kb)
Additional file 3: Causes of hospital admission in hyponatremic and normonatremic patients. Causes of hospital admission taking only into account the first admission per patient. (DOCX 68 kb)

## Abbreviations

AIDS: Acquired immunodeficiency syndrome
ARC: AIDS-related complex
cART: Combined antiretroviral therapy
CI: Confidence interval
HIV: Human immunodeficiency virus
IQR: Interquartile range
vs: Versus
WHO: World Health Organization
