# Supplementary material for: Hyponatremia is a marker of disease severity in HIV-infected patients: a retrospective cohort study
Source: BMC Infect Dis. 2017 Jan 26;17:98. doi: 10.1186/s12879-017-2191-5 (PMC5267411; doi:10.1186/s12879-017-2191-5)
Supplement: Additional file 1: — Main characteristics of patients lost to follow-up. Clinical and socio-demographic factors in patients lost to follow-up compared with patients not lost to follow-up. 1mean ± standard deviation; 2median (interquartile range); 3excluding patients hospitalized at first contact. (DOCX 92 kb) [file 12879_2017_2191_MOESM1_ESM.docx]

| **Characteristics** | **Total (N=1196)** | **Normal follow-up (N=782)** | **Loss to follow-up (N=414)** | **P-value** |
| --- | --- | --- | --- | --- |
| Age (years)^1^ | 36.8±10.7 | 37.6±10.9 | 35.4±10.3 | 0.0005 |
| Female gender (n) | 445 (37.2%) | 273 (34.9%) | 172 (41.5%) | 0.028 |
| African ethnicity (n) | 612 (51.2%) | 363 (46.4%) | 249 (60.1%) | <0.0001 |
| Homo-bisexual orientation (n) | 395 (33.0%) | 297 (38.0%) | 98 (23.7%) | <0.0001 |
| Natremia (mmol/l)^2^ | 139.0 (136.0-141.0) | 139.0 (137.0-141.0) | 138.0 (136.0-140.0) | <0.0001 |
| Na < 135 mmol/l (n) | 177 (14.8%) | 106 (13.5%) | 71 (17.1%) | 0.104 |
| Follow-up (months)^2^ | 36.0 (9.0-80.0) | 54.0 (23.0-102.0) | 9.0 (1.0-39.0) | <0.0001 |
| Mean number of hospitalizations per patient (n)^1^ | 2.2±4.9 | 2.5±4.5 | 1.6±5.5 | <0.0001 |
| Hospitalization at first contact (n) | 332 (27.7%) | 196 (25.1%) | 136 (32.8%) | 0.005 |
| Median time to first hospitalization (months)^2,3^ | 12.0 (2.0-28.0) | 14.0 (2.0-30.0) | 6.0 (1.0-24.0) | 0.007 |
| Acquired immunodeficiency syndrome (AIDS) (n) | 215 (18.0%) | 145 (18.5%) | 70 (16.9%) | 0.527 |
| CD4 cell count (/μl)^1^ | 371.8±275.3 | 369.1±274.1 | 376.8±277.8 | 0.669 |
| CD4 cell count < 350/μl (n) | 611 (51.1%) | 402 (51.7%) | 209 (51.3%) | 0.951 |
| CD4 nadir (/μl)^1^ | 362.8±267.6 | 360.5±264.3 | 367.2±274.1 | 0.801 |
| CD4 nadir < 200/μl (n) | 360 (30.1%) | 240 (31.4%) | 120 (29.6%) | 0.549 |
| HIV viral load (copies/ml)^2^ | 71600 (13500-100000) | 79800 (16600-121000) | 52400 (8910-100000) | 0.0012 |
| HIV viral load > 100 000 copies/ml (n) | 465 (38.9%) | 322 (43.4%) | 143 (37.3%) | 0.055 |
| Hepatitis B (n) | 69 (5.8%) | 43 (6.2%) | 26 (7.4%) | 0.511 |
| Hepatitis C (n) | 69 (5.8%) | 31 (4.3%) | 38 (10.9%) | <0.0001 |
| Fib 4 score > 3.25 (n) | 77 (6.4%) | 48 (6.1%) | 29 (7.1%) | 0.536 |
| Anemia (n) | 597 (50%) | 385 (49.3%) | 212 (51.3%) | 0.543 |
| Hyperlipidemia (n) | 418 (34.9%) | 299 (38.2%) | 119 (28.7%) | 0.0011 |
| Mean triglyceridemia^1^ | 111.1±68.5 | 113.3±72.2 | 106.5±59.7 | 0.364 |
| Diabetes mellitus (n) | 52 (4.3%) | 44 (5.6%) | 8 (1.9%) | 0.0026 |
| Antihypertensive drugs (n) | 39 (3.3%) | 34 (4.3%) | 5 (1.2%) | 0.0031 |
| ^1^mean±standard deviation |  |  |  |  |
| ^2^median (interquartile range) |  |  |  |  |
| ^3^excluding patients hospitalized at first contact |  |  |  |  |
