# Supplementary material for: Hyponatremia is a marker of disease severity in HIV-infected patients: a retrospective cohort study
Source: BMC Infect Dis. 2017 Jan 26;17:98. doi: 10.1186/s12879-017-2191-5 (PMC5267411; doi:10.1186/s12879-017-2191-5)
Supplement: Additional file 2: — Risk factors for mortality of patients with CD4 < 200/μl in univariate/multivariate Cox’s proportional hazard models. Association of hyponatremia with mortality in a restricted population of patients who had advanced HIV disease with a CD4 count < 200/μl. (DOCX 52 kb) [file 12879_2017_2191_MOESM2_ESM.docx]

|  | **Univariate Model** | | **Multivariate Model** | |
| --- | --- | --- | --- | --- |
| **Risk factors** | **Hazard ratio (95% Confidence interval)** | **P-value** | **Hazard ratio (95% Confidence interval)** | **P-value** |
| Age < 35 years | 0.86 (0.45-1.64) | 0.746 |  |  |
| Female gender | 1.37 (0.74-2.54) | 0.348 |  |  |
| African ethnicity | 0.87 (0.46-1.65) | 0.740 |  |  |
| Homo-bisexuel orientation | 0.59 (0.24-1.46) | 0.319 |  |  |
| Natremia < 135mmol/l | 1.92 (1.02-3.60) | 0.017 | 1.15 (0.63-2.09) | 0.646 |
| AIDS | 5.44 (2.54-11.64) | <0.0001 | 4.51 (2.13-9.57) | <0.0001 |
| HIV viral load > 100 000 copies/ml | 1.37 (0.61-3.11) | 0.560 |  |  |
| Hepatitis B | 0.24 (0.034-1.85) | 0.223 |  |  |
| Hepatitis C | 1.47 (0.47-4.55) | 0.516 |  |  |
| Fib4 score > 3.25 | 1.64 (0.74-3.65) | 0.250 |  |  |
| Anemia | 0.72 (0.36-1.45) | 0.353 |  |  |
| Hyperlipidemia | 0.84 (0.45-1.60) | 0.635 |  |  |
| Diabetes mellitus | 1.96 (0.62-6.23) | 0.275 |  |  |
| Inclusion period 1998-2004 | 0.61 (0.33-1.14) | 0.157 |  |  |
