# Supplementary material for: Hyponatremia is a marker of disease severity in HIV-infected patients: a retrospective cohort study
Source: BMC Infect Dis. 2017 Jan 26;17:98. doi: 10.1186/s12879-017-2191-5 (PMC5267411; doi:10.1186/s12879-017-2191-5)
Supplement: Additional file 3: — Causes of hospital admission in hyponatremic and normonatremic patients. Causes of hospital admission taking only into account the first admission per patient. (DOCX 68 kb) [file 12879_2017_2191_MOESM3_ESM.docx]

| **Causes of hospital admission** | **Total number of patients (N=1196)** | **Na < 135 mmol/l (N=177)** | **Na ≥ 135 mmol/l (N=1019)** | **P-value** |
| --- | --- | --- | --- | --- |
| AIDS-related | 92 (7.7%) | 39 (22.0%) | 53 (5.2%) | <0.0001 |
| Malnutrition/wasting | 14 (1.17%) | 3 (1.69%) | 11 (1.08%) | 0.448 |
| Parasitic infections | 59 (4.9%) | 15 (8.5%) | 44 (4.3%) | 0.024 |
| Bacterial infections | 207 (17.3%) | 72 (40.7%) | 135 (13.2%) | <0.0001 |
| Malignancies | 55 (4.6%) | 18 (10.2%) | 37 (3.6%) | 0.0006 |
| Drug toxicities | 42 (3.5%) | 11 (6.2%) | 31 (3.0%) | 0.045 |
| Neurological | 68 (5.7%) | 14 (7.9%) | 54 (5.3%) | 0.162 |
| Cardiovascular | 17 (1.4%) | 4 (2.3%) | 13 (1.3%) | 0.300 |
| Renal | 28 (2.3%) | 7 (3.9%) | 21 (2.1%) | 0.170 |
| Endocrine/metabolic | 13 (1.1%) | 6 (3.4%) | 7 (0.7%) | 0.007 |
| Haematological | 26 (2.2%) | 6 (3.4%) | 20 (2.0%) | 0.258 |
| Respiratory | 118 (9.9%) | 43 (24.3%) | 75 (7.4%) | <0.0001 |
| Digestive | 81 (6.8%) | 19 (10.7%) | 62 (6.1%) | 0.033 |
| Liver | 15 (1.2%) | 3 (1.7%) | 12 (1.2%) | 0.476 |
| Viral | 90 (7.5%) | 18 (10.2%) | 72 (7.1%) | 0.164 |
| Skin/soft tissue | 39 (3.3%) | 9 (5.1%) | 30 (2.9%) | 0.165 |
| Psychiatric disorders | 12 (1.0%) | 3 (1.7%) | 9 (0.9%) | 0.402 |
| Gynaecological | 99 (8.3%) | 23 (13.0%) | 76 (7.5%) | 0.018 |
| Other | 131 (10.9%) | 26 (14.7%) | 105 (10.3%) | 0.090 |
| Unknown | 250 (20.9%) | 52 (29.4%) | 198 (19.4%) | 0.004 |
